# Effectiveness and safety of different doses of febuxostat compared with allopurinol in the treatment of hyperuricemia: a meta-analysis of randomized controlled trials

**DOI:** 10.1186/s40360-023-00723-5

**Published:** 2023-12-14

**Authors:** Hong Xie, Nan Hu, Ting Pan, Jun-Cai Wu, Miao Yu, Deng-Chao Wang

**Affiliations:** 1 Department of General Medicine, Zigong Fourth People’s Hospital, 643000 Zigong, Sichuan China; 2 Department of General Surgery, Zigong Fourth People’s Hospital, 19 Tanmulin Road, 643000 Zigong, Sichuan China; 3 Department of Basic Medicine, Sichuan Vocational College of Health and Rehabilitation, 643000 Zigong, Sichuan China

**Keywords:** Febuxostat, Allopurinol, Hyperuricemia, Meta-analysis

## Abstract

**Background:**

The prevalence of hyperuricemia has increased steadily with the continuous improvement of living standards. Some studies have reported the clinical effectiveness and safety of different doses of febuxostat in comparison with allopurinol in hyperuricemia treatment, but the sample sizes of the studies have been small, and the results have been inconsistent. We designed this meta-analysis to evaluate the effectiveness and safety of different doses of febuxostat compared with allopurinol in the treatment of hyperuricemia.

**Methods:**

The Cochrane Library, Embase, PubMed, Web of Science and ClinicalTrials.gov databases were searched to identify randomized controlled trials (RCTs) comparing the use of febuxostat and allopurinol for the treatment of hyperuricemia. The effectiveness and safety of different doses of febuxostat and allopurinol in treating hyperuricemia were assessed using meta-analysis.

**Results:**

A total of 11 randomized controlled trials were included in the meta-analysis. The results of the meta-analysis showed that the percentage of patients achieving serum uric acid levels of 6.0 mg/dL or less was higher among patients taking febuxostat (80 mg/d) than among patients taking allopurinol (200–300 mg/d) [RR = 1.79, 95% CI (1.55, 2.08), *P* < 0.00001]. However, there was no statistically significant difference in the percentage of patients achieving serum uric acid levels of 6.0 mg/dL or less between febuxostat (40 mg/d) and allopurinol (200–300 mg/d) [RR = 1.10, 95% CI (0.93, 1.31), *P* = 0.25]. There was also no statistically significant difference in the incidence of gout between the febuxostat (40 mg/d) and allopurinol (200–300 mg/d) [RR = 0.97, 95% CI (0.64, 1.49), *P* = 0.91] or between the febuxostat (80 mg/d) and allopurinol (200–300 mg/d) [RR = 1.13, 95% CI (0.81, 1.58), P = 0.48].No significant difference in the incidence of major adverse reactions as observed between the febuxostat (40 mg/d) and allopurinol (200–300 mg/d) [RR = 1.16; 95% CI (0.43, 3.16), *P* = 0.77] or between the febuxostat (80 mg/d) and allopurinol (200–300 mg/d) [RR = 1.06; 95% CI (0.79, 1.42), *P* = 0.70]. The incidence of adverse cardiovascular events did not differ significantly between the febuxostat (40 mg/d) and allopurinol (200–300 mg/d) [RR = 1.30; 95% CI (0.57, 2.95), *P* = 0.53] or between the febuxostat (80 mg/d) and allopurinol (200–300 mg/d) [RR = 1.79; 95% CI (0.74, 4.32), *P* = 0.20].

**Conclusions:**

Febuxostat (80 mg/d) was associated with a higher percentage of patients achieving serum uric acid levels of 6.0 mg/dL or less than allopurinol (200–300 mg/d), however, febuxostat (80 mg/d) did not exhibit better efficacy in reducing the incidence of gout. More attention should be devoted to the adverse reactions caused by an increase in febuxostat doses.

**Supplementary Information:**

The online version contains supplementary material available at 10.1186/s40360-023-00723-5.

## Introduction

The prevalence of hyperuricemia has increased steadily with the continuous improvement of living standards [1], and its prevalence is higher in men than in women [2]. Hyperuricemia is also an independent risk factor for metabolic diseases (diabetes, metabolic syndrome, hyperlipidemia, etc.), chronic kidney disease, cardiovascular disease, and stroke [3–5]. Gout is a crystalline-associated arthropathy caused by monosodium urate deposition and belongs to the category of metabolic diseases [6], and it has been reported that hyperuricemia is closely associated with gout [7]. At present, it is believed that the main causes of hyperuricemia are abnormal purine metabolism and decreased excretion of uric acid in the body [8, 9]. The level of serum uric acid (SUA) is mainly affected by two factors: the synthesis of uric acid and the other is the excretion of uric acid. Currently, commonly used drugs used in clinical practice, such as probenecid, help to lower the uric acid by increasing the excretion of uric acid, whereas other drugs, such as allopurinol and febuxostat, inhibit its synthesis [10–12]. The inhibition of uric acid synthesis is essential for patients with hyperuricemia. Allopurinol and its metabolite, i.e., oxypurinol, reduce uric acid synthesis by suppressing the activity of xanthine oxidase, an enzyme that converts hypoxanthine to xanthine and then converts xanthine to uric acid [13]. However, allopurinol is related to several adverse reactions; for example, when the glomerular filtration rate decreases, the risk of toxicity increases, in turn leading bone marrow depression, hepatotoxicity, and a risk of hypersensitivity syndrome [14].

Febuxostat is a relatively new type of urate-lowering drug, that can selectively inhibit xanthine oxidase and improve purine metabolism. Febuxostat is primarily metabolized in the liver and excreted by both renal and intestinal channels after oral administration, which is very effective in lowering uric acid and enhancing renal protection compared with other drugs [15–17]. Some studies have reported the clinical effectiveness and safety of different doses of febuxostat in comparison with allopurinol in hyperuricemia treatment, but the sample size included in a single study is small, and the results of different studies are not consistent [18–28]. Several meta-analyses have compared the efficacy and safety of the two drugs; they have primarily focused on the overall comparison of the medications and overlooking the significance of dosage in terms of treatment effectiveness and safety [29–31]. Based on that, this meta-analysis thoroughly assessed the percentage of patients achieving serum uric acid levels of 6.0 mg/dL or less, the incidence of gout, the incidence of serious adverse reactions and the incidence of adverse cardiovascular reactions associated with febuxostat (40 and 80 mg/d) and allopurinol (200–300 mg/d) administered to patients with hyperuricemia. Additionally, this study employs the GRADE (Grading of Recommendations Assessment, Development, and Evaluation) system to assess the outcome measures, thus providing evidence-based recommendations for clinical treatment.

## Materials and methods

We conducted and reported this systematic review in accordance with the Preferred Reporting Items for Systematic Reviews and Meta-Analyses for Protocols guidelines [32]. The registration number is INPLASY2022110017. The detailed information regarding registration can be found on the following website: https://inplasy.com/inplasy-2022-11-0017/.

### Inclusion and exclusion criteria

#### Inclusion criteria

The following inclusion criteria were used in this study: (1) Participants: Patients with hyperuricemia, serum uric acid ≥ 405 µmol/L (6.8 mg/dL), and age ≥ 18 years old. (2) Interventions: Patients included in the experimental group were treated using febuxostat (at a dose of 40 mg/day or 80 mg/day), while those in the control group were treated with allopurinol, with no restrictions on the duration of treatment and follow-up. (3) Research type: Randomized controlled trials published in the English language. (4) Outcome measures: primary outcome measure : percentage of patients achieving serum uric acid levels of 6.0 mg/dL or less; secondary outcome measures: the incidence of gout (the frequency of gout attacks during the study duration), incidence of serious adverse reactions (a serious adverse reaction was a reaction that was life-threatening or resulted in death, hospitalization or prolongation of hospitalization, persistent or significant disability or incapacity, such as chest pain, coronary artery disease, myocardial infarction, atrial fibrillation, thrombocytopenia, or pleuritic pain), and incidence of adverse cardiovascular reactions (e.g., cardiovascular death, myocardial infarction, angina, nonfatal stroke).

#### Exclusion criteria

The following exclusion criteria were used: (1) nonrandomized controlled trials; (2) duplicate publications; (3) unable to extract outcome measures; (4) literature cannot be entirely acquired; (5) allopurinol was not used in the control group for intervention; (6) patients receiving treatment for hyperuricemia with medications other than febuxostat or allopurinol; and (7) patients with moderate or severe liver impairment or severe renal impairment.

### Search strategy

The Cochrane Library, Embase, PubMed, Web of Science and ClinicalTrials.gov databases were searched from inception to August 31, 2022. The search terms were febuxostat, allopurinol, gout, and hyperuricemia. A combination of MeSH terms and entry terms was used for search purposes. Furthermore, the references lists of the included studies were manually searched to identify additional eligible studies.

### Literature screening and data extraction

Based on the inclusion and exclusion criteria described above, two researchers independently screened the literature. Disagreements were resolved by discussion and consensus with a third researcher. Missing data were obtained by contacting the original author. The titles and abstracts were screened first to exclude unrelated literature, and then, the full texts were read further to determine final inclusion. The following data were extracted: (1) first author and publication year; (2) country; (3) sample size; (4) age; (5) body mass index (BMI); (6) baseline serum urate concentration; (7) time of follow-up; (8) previous urate lowering therapy; (9) renal impairment; (10) race; (11) coexisting conditions; and (12) Outcome measures.

### Quality evaluation

Two researchers assessed the risk of bias across all studies independently and cross-validated their results. Disagreements were resolved via discussion. The quality of the included RCTs was evaluated with the risk of bias assessment tool recommended by the Cochrane Handbook of Systematic Reviewers 5.3. The following seven aspects were assessed: (1) method of randomization; (2) concealment of allocation scheme; (3) double blinding parameters of experimenters and participants; (4) blinding assessment of the results; (5) completeness of the resulting data; (6) selective reporting of results; and (7) other sources of bias. Each study was rated as “low risk of bias”, “unclear”, and “high risk of bias” [33].

### Statistical analysis

The present meta-analysis was conducted using RevMan 5.3 software offered by the Cochrane Collaboration. The relative risk ratio (RR) was used as the effect size for dichotomous variables, and their pooled effect size and 95% confidence interval (CI) were also calculated. Heterogeneity noted across all study results was evaluated using the χ2 test, and the size of heterogeneity was quantitatively determined in combination with I^2^. If there was no statistical heterogeneity across the study results (*P* > 0.10, I^2^ ≤ 50%), a fixed effects model was used for the meta-analysis. However, when there was statistical heterogeneity across the study results (I^2^ > 50%), a random effects model was used for the meta-analysis [34]. Only the RCTs with considerable clinical heterogeneity were subjected to sensitivity analysis and subgroup analysis. When there were ≥ 10 for a relevant study indicator, publication bias was evaluated by examining funnel plot [35].

### Evaluation of evidence quality

Following the GRADE criteria, GRADEprofiler 3.6 was utilized to assess the quality of evidence for each outcome indicator. Based on five aspects, including risk of bias in studies, inconsistency, indirectness, imprecision, and publication bias, the outcome measures were classified into four levels: high, moderate, low, and very low [36].

## Results

### Literature search results

**Fig. 1:**
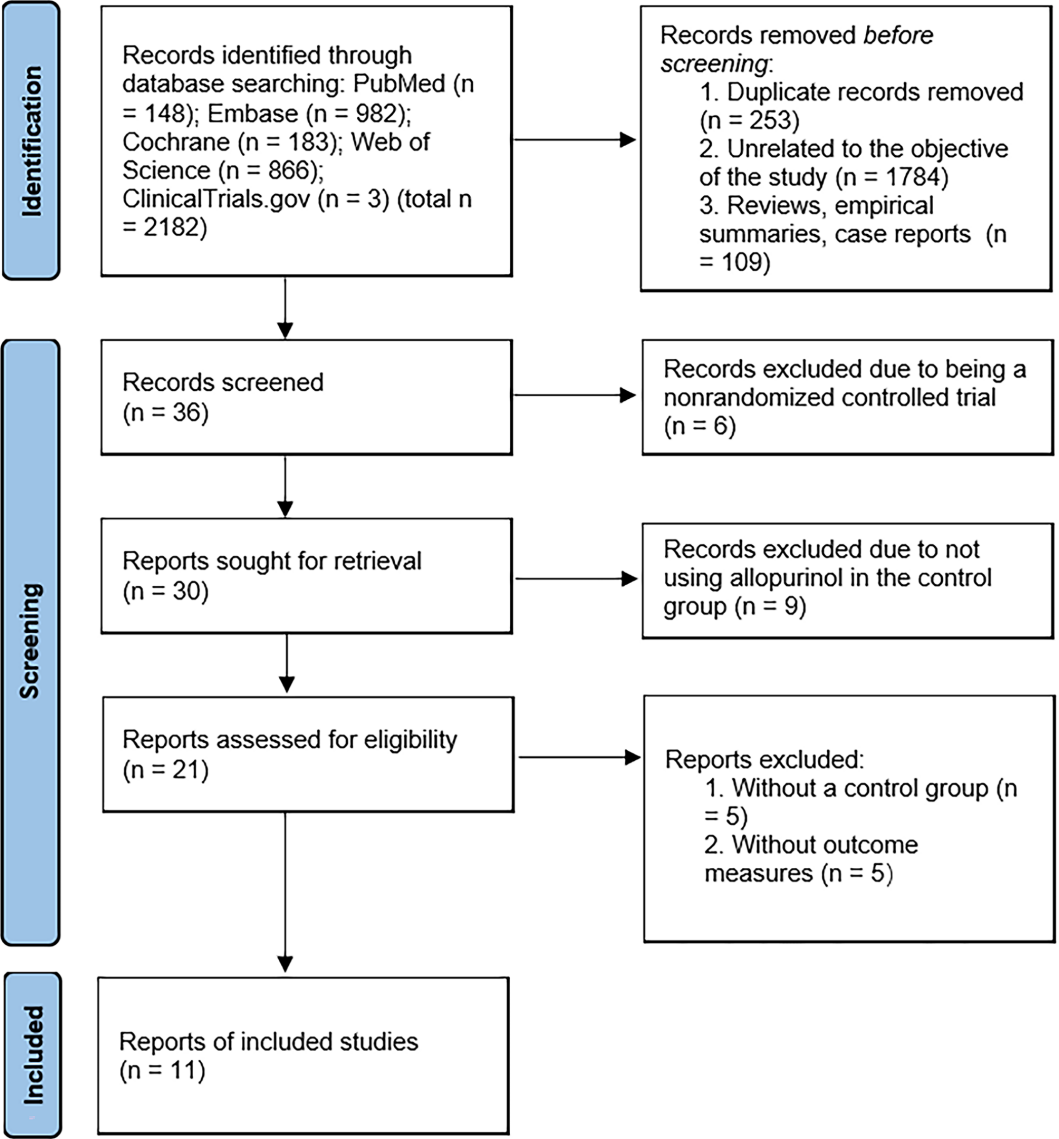
PRISMA flow diagram depicting the selection process

In this study, 2182 records were initially retrieved from the database. A total of 253 duplicate records were excluded by reading titles and abstracts, 1784 records were excluded due to being unrelated to the objective of the study; and 109 records were excluded due to being reviews, empirical summaries or case reports. The remaining 36 records were rescreened by reading full texts. Six records were excluded due to being nonrandomized controlled studies, 9 records were excluded due to not using allopurinol in the control group, 5 records were excluded due to not having control group, and 5 were excluded due to not using the relevant outcome measures. Ultimately, 11 records were included [18–28], and this complete screening process is detailed in Fig. 1. Tables 1 and 2 presents the characteristics of the studies included in this meta-analysis.

**Table 1:** Characteristics of the studies included in this meta-analysis

First author and				Sample size		BMI	Baseline serum urate		Outcome
publication year	Country	Group	Intervention	(M/F)	Age (year)	(kg/m^2^)	concentration	Time of follow-up	indicators
Becker 2005 [18]	USA	Febuxostat	Febuxostat 80 mg/day	243/13	51.8 ± 11.7	32.7 ± 6.1	9.8 ± 1.2 mg/dL	52 w	①②③
		Allopurinol	Allopurinol 300 mg/day	243/10	51.6 ± 12.6	32.6 ± 6.1	9.9 ± 1.2 mg/dL		
Becker 2009 [19]	USA	Febuxostat	Febuxostat 80 mg/day	649(Na/Na)	51.4 ± 11.9	32.3 ± 5.7	9.8 ± 1.2 mg/dL	3 y	①③④
		Allopurinol	Allopurinol 300 mg/day	145 (Na/Na)	51.0 ± 11.3	33.8 ± 6.7	9.8 ± 1.1 mg/dL		
Becker 2010 [20]	USA	Febuxostat	Febuxostat 40 mg/day	722/35	52.5 ± 11.6	32.9 ± 6.3	9.6 ± 1.1 mg/dL	28 w	①②④
			Febuxostat 80 mg/day	710/46	53.0 ± 11.7	32.9 ± 6.3	9.6 ± 1.2 mg/dL		
		Allopurinol	Allopurinol 200/300 mg/day	709/47	52.9 ± 11.7	32.7 ± 6.2	9.5 ± 1.1 mg/dL		
Huang 2014 [21]	China	Febuxostat	Febuxostat 40 mg/day	167/5	46.42 ± 10.9	25.6 ± 2.8	9.8 ± 1.3 mg/dL	28 w	③
			Febuxostat 80 mg/day	169/3	46.4 ± 10.9	25.2 ± 2.6	9.9 ± 1.3 mg/dL		
		Allopurinol	Allopurinol 300 mg/day	168/4	46.4 ± 10.9	25.4 ± 2.5	9.9 ± 1.3 mg/dL		
Kamatani 2011 [22]	Japan	Febuxostat	Febuxostat 40 mg/day	10/0	56 ± 8.2	Na	8.6 ± 0.7 mg/dL	16 w	①
		Allopurinol	Allopurinol 300 mg/day	19/0	51.3 ± 12	Na	8.3 ± 1.1 mg/dL		
Mackenzie 2020 [23]	UK	Febuxostat	Febuxostat 80 mg/day	2619/444	71.0 ± 6.4	31.0 ± 5.1	0.297 mmol/L	1467 (1029–2052) d	③
		Allopurinol	Allopurinol 200/300 mg/day	2606/459	70.9 ± 6.5	31.2 ± 5.3	0.297 mmol/L		
Nakagomi 2015 [24]	Japan	Febuxostat	Febuxostat 40 mg/day	22/9	69.3 ± 10.0	23.6 ± 2.4	9.4 ± 0.5 mg/dL	23.0 (13–47) m	③
		Allopurinol	Allopurinol 300 mg/day	18/12	71.8 ± 8.0	23.1 ± 3.1	9.3 ± 0.5 mg/dL		
Schumacher 2008 [25]	USA	Febuxostat	Febuxostat 80 mg/day	251/16	51 ± 12	33 ± 6	> 8 mg/dL	28 w	①③④
		Allopurinol	Allopurinol 300 mg/day	249/19	52 ± 12	33 ± 6	> 8 mg/dL		
Xu 2015 [26]	China	Febuxostat	Febuxostat 40 mg/day	158/2	45.5 ± 11.9	25.3 ± 2.7	560.8 ± 73.3 umol/L	24 w	①②③④
			Febuxostat 80 mg/day	146/12	48.2 ± 12.0	25.1 ± 2.6	565.1 ± 75.5 umol/L		
		Allopurinol	Allopurinol 300 mg/day	149/10	46.6 ± 10.7	25.4 ± 3.3	74.2 ± 77.8 umol/L		
Yu 2016 [27]	Taiwan	Febuxostat	Febuxostat 80 mg/day	53/1	46.0 ± 11.0	26.8 ± 3.7	> 8 mg/dL	12 w	①③
		Allopurinol	Allopurinol 300 mg/day	53/2	45.2 ± 12.0	27.8 ± 4.2	> 8 mg/dL		
Zhang 2019 [28]	China	Febuxostat	Febuxostat 40 mg/day	181/1	46.5 ± 11.9	26.1 ± 3.2	9.6 ± 1.5 mg/dL	24 w	①③
			Febuxostat 80 mg/day	184/4	46.5 ± 11.1	25.7 ± 3.2	9.6 ± 1.5 mg/dL		
		Allopurinol	Allopurinol 300 mg/day	182/2	46.5 ± 11.1	26.0 ± 3.4	9.8 ± 1.4 mg/dL		

BMI, Body Mass Index; d, day; F, female; M, male; m, month; Na, not available; w, week; y, year. Allopurinol 200/300 mg/day, patient with normal renal function or mild renal impairment received 300 mg daily, and those with moderate renal impairment received 200 mg daily. ① Percentage of patients achieving serum uric acid levels 6.0 mg/dL or less; ② the incidence of gout; ③ incidence of serious adverse reactions; ④ incidence of adverse cardiovascular reactions

**Table 2:** Characteristics of the studies included in this meta-analysis

First author and			Previous urate	Renal		
publication year	Group	Intervention	lowering therapy	impairment	Race	Coexisting conditions
Becker 2005 [18]	Febuxostat	Febuxostat 80 mg/d	112	90	White 193; Black 24; Hispanic 22; Asian 10; Other 7	Hypercholesterolemia 19; Hyperlipidemia 90; Hypertension 106; Obesity 166; Urolithiasis 49; Metabolic syndrome 19
	Allopurinol	Allopurinol 300 mg/d	113	81	White 195; Black 18; Hispanic 19; Asian 6; Other 15	Hypercholesterolemia 19; Hyperlipidemia 90; Hypertension 106; Obesity 166; Urolithiasis 49; Metabolic syndrome 19
Becker 2009 [19]	Febuxostat	Febuxostat 80 mg/d	Na	13	Asian 19; Black or African American 51; White 519; Hispanic or Latino 40; Other 20	Cardiovascular disease 71; Congestive heart failure 11; Diabetes 46; Hypercholesterolemia 48; Hyperlipidemia 229; Hypertension 295
	Allopurinol	Allopurinol 300 mg/d	Na	1	Asian 5; Black or African American 15; White 110; Hispanic or Latino 11; Other 4	Cardiovascular disease14; Congestive heart failure 0; Diabetes 12; Hypercholesterolemia 9; Hyperlipidemia 47; Hypertension 73
Becker 2010 [20]	Febuxostat	Febuxostat 40 mg/d	Na	479	American Indian or Alaska Native 6; Asian 26; Black or African American 83; Native Hawaiian or Other Pacific Islander 11; White 620; Other 11	Cardiovascular Disease 421; Diabetes 89; Hypercholesterolemia 52; Hyperlipidemia 299
		Febuxostat 80 mg/d	Na	503	American Indian or Alaska Native 10; Asian 25; Black or African American 78; Native Hawaiian or Other Pacific Islander 10; White 618; Other 15	Cardiovascular Disease 440; Diabetes113; Hypercholesterolemia 53; Hyperlipidemia308
	Allopurinol	Allopurinol 200/300 mg/d	Na	501	American Indian or Alaska Native 6; Asian 37; Black or African American 67; Native Hawaiian or Other Pacific Islander 11; White 625; Other 8	Cardiovascular Disease 436; Diabetes110; Hypercholesterolemia 57; Hyperlipidemia 335
Huang 2014 [21]	Febuxostat	Febuxostat 40 mg/d	Na	4	NA	Hypertension 54; Hyperlipidemia 6; Endocrine system, including diabetes 14; Hyperthyroidism 3; Digestive system 8; Cardiovascular disease 57
		Febuxostat 80 mg/d	Na	5	NA	Hypertension 45; Hyperlipidemia 5; Endocrine system, including diabetes 9; Hyperthyroidism 0; Digestive system 7; Cardiovascular disease 47
	Allopurinol	Allopurinol 300 mg/d	Na	6	NA	Hypertension 44; Hyperlipidemia 2; Endocrine system, including diabetes 10; Hyperthyroidism 4; Digestive system 14; Cardiovascular disease 45
Kamatani 2011 [22]	Febuxostat	Febuxostat 40 mg/d	8	0	NA	Hypertension 6; Hyperlipidemia 2; Diabetes 1; Hepatic disease 1
	Allopurinol	Allopurinol 300 mg/d	17	0	NA	Hypertension 7; Hyperlipidemia 9; Diabetes 1; Hepatic disease 0
Mackenzie 2020 [23]	Febuxostat	Febuxostat 80 mg/d	Na	504	White 3034; Asian 11; Afro-Caribbean 10; Oriental 2; Other 6	Cardiovascular history 5339; Asthma 334; Chronic obstructive pulmonary disease 211; Diabetes 661
	Allopurinol	Allopurinol 200/300 mg/d	Na	483	White 3036; Asian 14; Afro-Caribbean 8; Oriental 1; Other 6	Cardiovascular history 5418; Asthma 358; Chronic obstructive pulmonary disease 228; Diabetes 719
Nakagomi 2015 [24]	Febuxostat	Febuxostat 40 mg/d	NA	NA	NA	Ischemic cardiomyopathy 20; Dilated cardiomyopathy 11; Hypertension 27; Diabetes 9; Dyslipidemia 30
	Allopurinol	Allopurinol 300 mg/d	NA	NA	NA	Ischemic cardiomyopathy 24; Dilated cardiomyopathy 6; Hypertension 30; Diabetes 12; Dyslipidemia 29
Schumacher 2008 [25]	Febuxostat	Febuxostat 80 mg/d	NA	9	White 200; Minority 67	Hypercholesterolemia 12; Hyperlipidemia 90; Hypertension 124; Cardiovascular disease 38
	Allopurinol	Allopurinol 300 mg/d	NA	10	White 206; Minority 62	Hypercholesterolemia 16; Hyperlipidemia 76; Hypertension 123; Cardiovascular disease 27
Xu 2015 [26]	Febuxostat	Febuxostat 40 mg/d	87	NA	NA	Hypertension 20; Diabetes 10; Hyperlipidemia 15; Cardiovascular disease 4
		Febuxostat 80 mg/d	79	NA	NA	Hypertension 32; Diabetes 5; Hyperlipidemia 13; Cardiovascular disease 2
	Allopurinol	Allopurinol 300 mg/d	83	NA	NA	Hypertension 22; Diabetes 9; Hyperlipidemia 11; Cardiovascular disease 4
Yu 2016 [27]	Febuxostat	Febuxostat 80 mg/d	13	NA	NA	Na
	Allopurinol	Allopurinol 300 mg/d	16	NA	NA	NA
Zhang 2019 [28]	Febuxostat	Febuxostat 40 mg/d	Na	Na	Na	Na
		Febuxostat 80 mg/d	Na	Na	Na	Na
	Allopurinol	Allopurinol 300 mg/d	Na	Na	Na	Na

Na, not available

### Results of literature quality evaluation

**Fig. 2:**
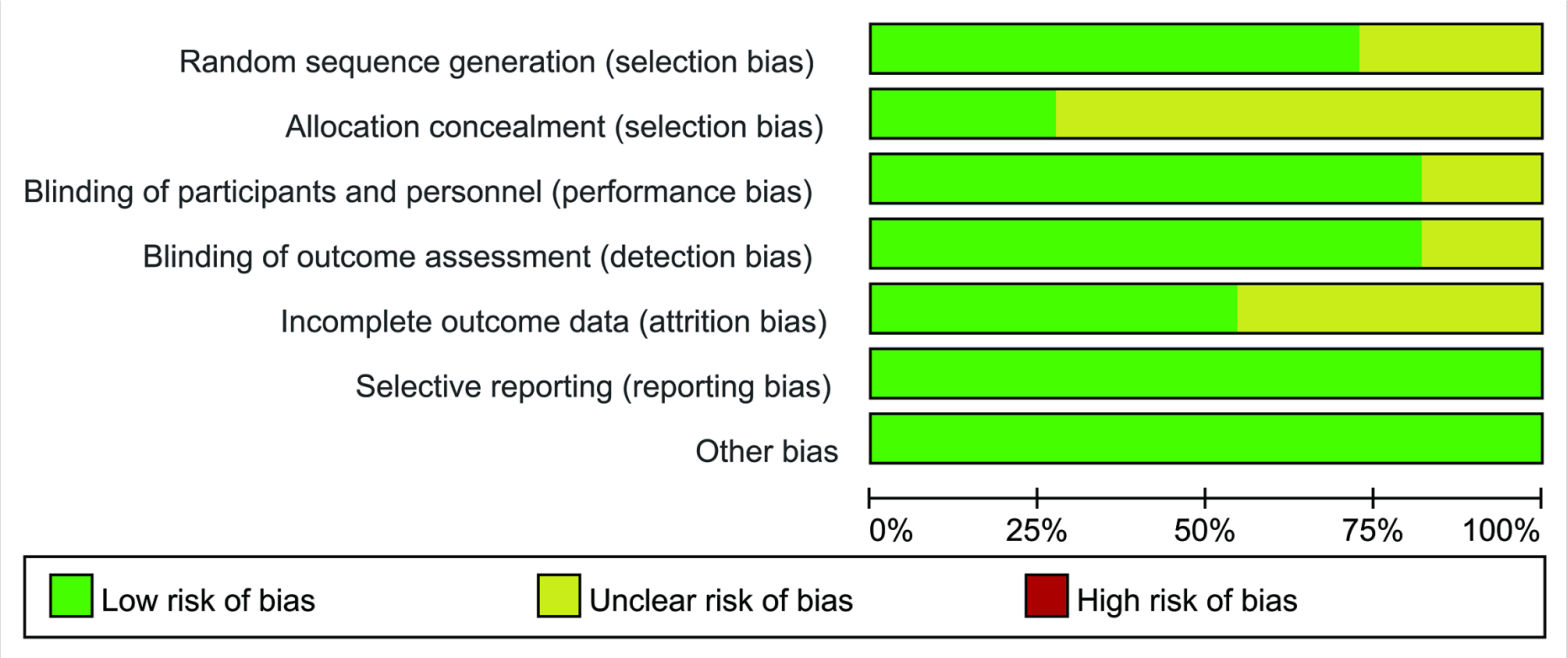
Risk of bias graph for the RCTs included in this meta-analysis

**Fig. 3:**
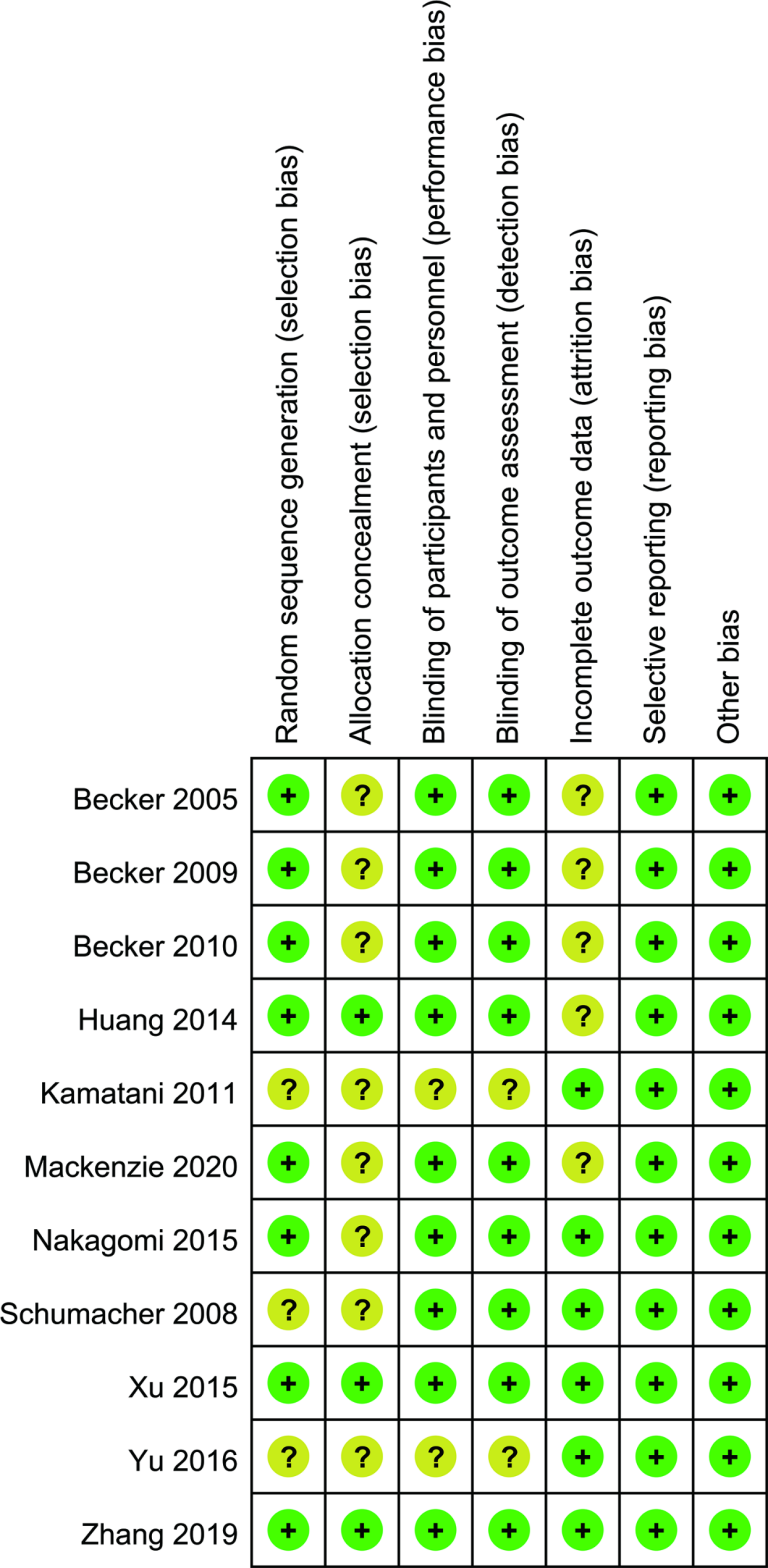
A summary of the risks of bias analysis for the RCTs included in the study

Eleven studies were all RCTs [18–28], of which 8 studies [18–21, 23, 24, 26, 28] used the correct random method, subjects were randomized using an interactive web response system (IWRS), SAS 9.1.3 software or computer-generated central randomization schedule. Three studies [21, 26, 28] used allocation scheme concealment. Nine studies [18–21, 23–26, 28] used blinding of study subjects and experimenters, 9 studies [18–21, 23–26, 28] blinded outcome assessors, 5 studies [18–21, 23] had the incomplete outcome data, and none studies [18–28] included a selective report of the results or other biases. Figures 2 and 3 present the details of the studies.

### Results of meta-analysis

#### Percentage of patients achieving serum uric acid levels 6.0 mg/dL or less

**Fig. 4:**
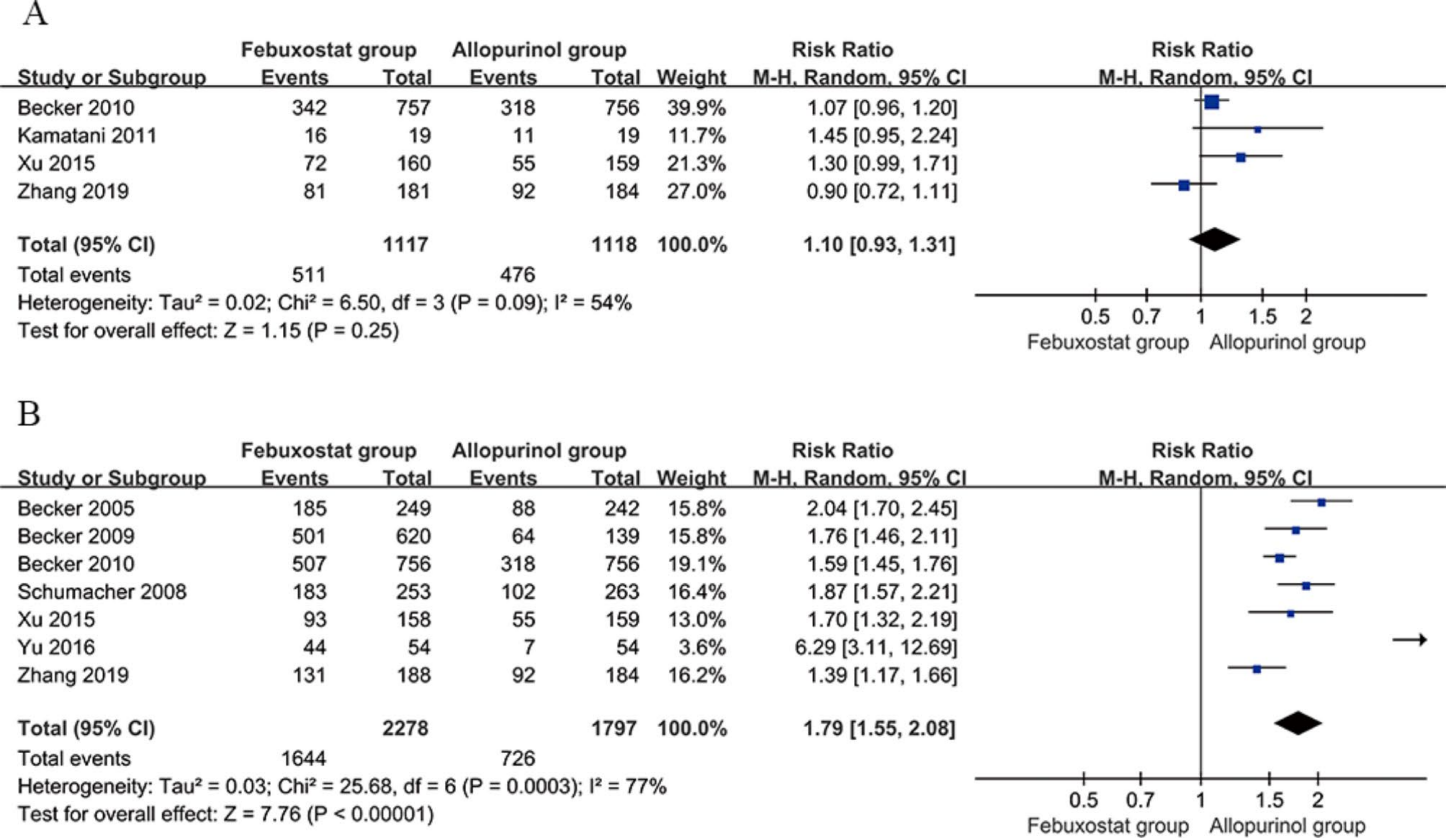
Forest plot comparing febuxostat and allopurinol in the percentage of patients achieving serum uric acid levels of 6.0 mg/dL or less. **a** Febuxostat (40 mg/d) versus allopurinol (200–300 mg/d). **b** Febuxostat (80 mg/d) versus allopurinol (200–300 mg/d). CI, confidence interval

Four studies [20, 22, 26, 28] compared the percentage of patients achieving serum uric acid levels of 6.0 mg/dL or less between the febuxostat group (40 mg/d) and the allopurinol group (200–300 mg/d). The percentage of patients achieving serum uric acid levels of 6.0 mg/dL or less in the febuxostat group (40 mg/d) was 511/1117 (45.7%), while in the allopurinol group (200–300 mg/d), it was 476/1118 (42.6%). There was significant heterogeneity among the studies (*P* = 0.09, I2 = 54%). Meta-analysis was conducted using a random effects model to pool the effect sizes, and the results indicated no statistically significant difference in the percentage of patients achieving serum uric acid levels of 6.0 mg/dL or less between the febuxostat group (40 mg/d) and the allopurinol group (200–300 mg/d) [RR = 1.10, 95% CI (0.93, 1.31), *P* = 0.25]. Sensitivity analysis was performed by sequentially excluding each included study, and the results showed no directional change in the pooled effect size after exclusion, suggesting that the results of this study are essentially stable. See Fig. 4A for details.

Seven studies [18–20, 25–28] compared the percentage of patients achieving serum uric acid levels of 6.0 mg/dL or less between the febuxostat group (80 mg/d) and the allopurinol group (200–300 mg/d). The percentage of patients achieving serum uric acid levels of 6.0 mg/dL or less in the febuxostat group (80 mg/d) was 1644/2278 (72.2%), while in the allopurinol group (200–300 mg/d), it was 726/1797 (40.4%). There was significant heterogeneity among the studies (*P* = 0.0003, I2 = 77%). Meta-analysis was conducted using a random effects model to pool the effect sizes, and the results indicated that the percentage of patients achieving serum uric acid levels of 6.0 mg/dL or less in the febuxostat group (80 mg/d) was higher than that in the allopurinol group (200–300 mg/d), and the difference was statistically significant [RR = 1.79, 95% CI (1.55, 2.08), *P* < 0.00001]. Sensitivity analysis was performed by sequentially excluding each included study, and the results showed no directional change in the pooled effect size after exclusion, suggesting that the results of this study are essentially stable. See Fig. 4B for details.

#### Incidence of gout

**Fig. 5:**
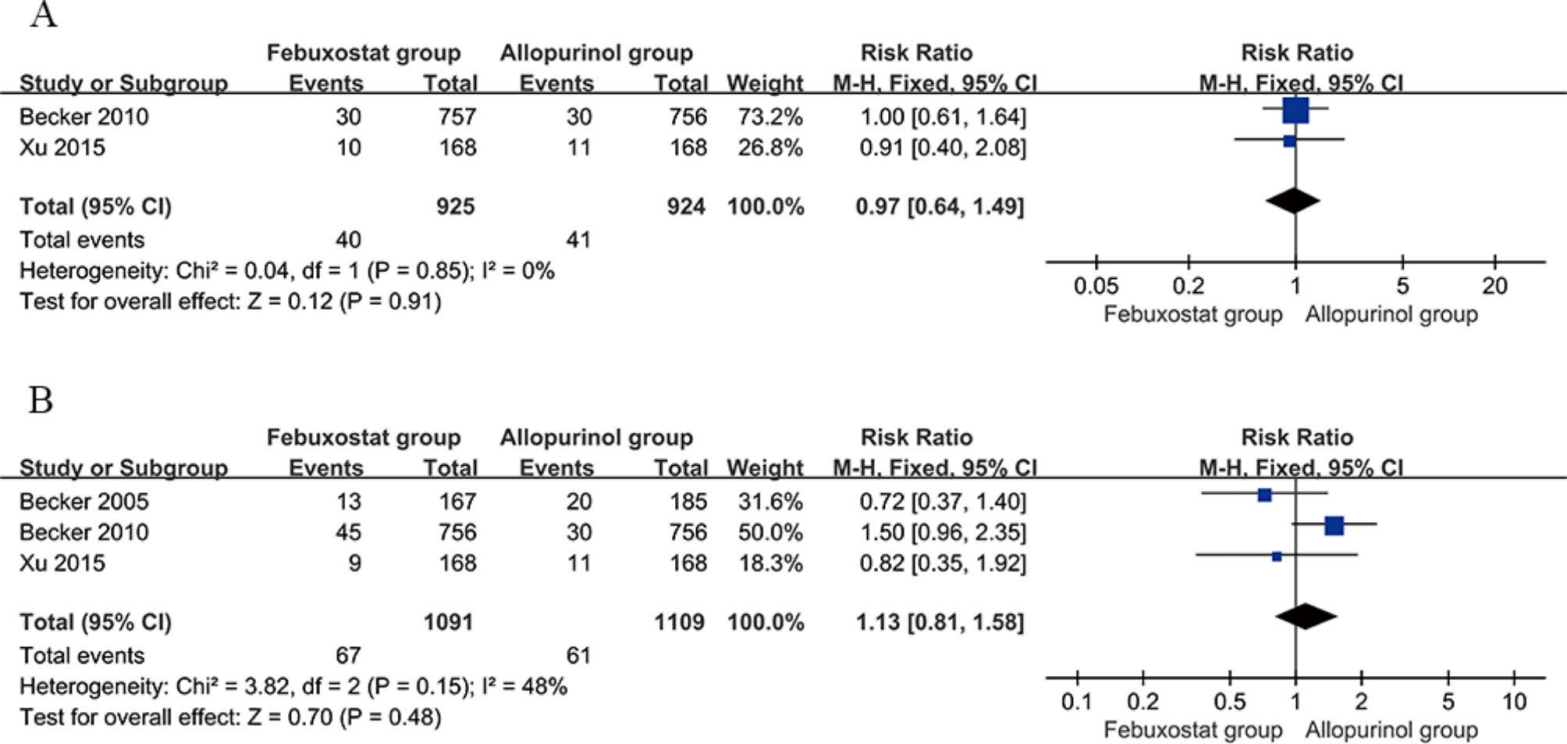
Forest plot comparing the incidence of gout between the febuxostat and allopurinol groups. **a** Febuxostat (40 mg/d) versus allopurinol (200–300 mg/d). **b** Febuxostat (80 mg/d) versus allopurinol (200–300 mg/d). CI, confidence interval

Two studies [20, 26] compared the incidence of gout between the febuxostat group (40 mg/d) and the allopurinol group (200–300 mg/d). The duration of follow-up for both studies ranged from 24 weeks to 28 weeks. The incidence of gout in the febuxostat group (40 mg/d) was 40/925 (4.3%), while in the allopurinol group (200–300 mg/d), it was 41/924 (4.4%). There was no significant heterogeneity among the studies (*P* = 0.85, I2 = 0%). Meta-analysis was conducted using a fixed effects model to pool the effect sizes, and the results indicated that there was no statistically significant difference in the incidence of gout between the febuxostat group (40 mg/d) and the allopurinol group (200–300 mg/d) [RR = 0.97, 95% CI (0.64, 1.49), *P* = 0.91]. See Fig. 5A for details.

Three studies [18, 20, 26] compared the incidence of gout between the febuxostat group (80 mg/d) and the allopurinol group (200–300 mg/d). The incidence of gout in the febuxostat group (80 mg/d) was 67/1091 (6.1%), while in the allopurinol group (200–300 mg/d), it was 61/1109 (5.5%). There was no significant heterogeneity among the studies (*P* = 0.15, I2 = 48%). Meta-analysis was conducted using a fixed effects model to pool the effect sizes, and the results indicated that there was no statistically significant difference in the incidence of gout between the febuxostat group (80 mg/d) and the allopurinol group (200–300 mg/d) [RR = 1.13, 95% CI (0.81, 1.58), *P* = 0.48]. See Fig. 5B for details.

#### Incidence of serious adverse reactions

**Fig. 6:**
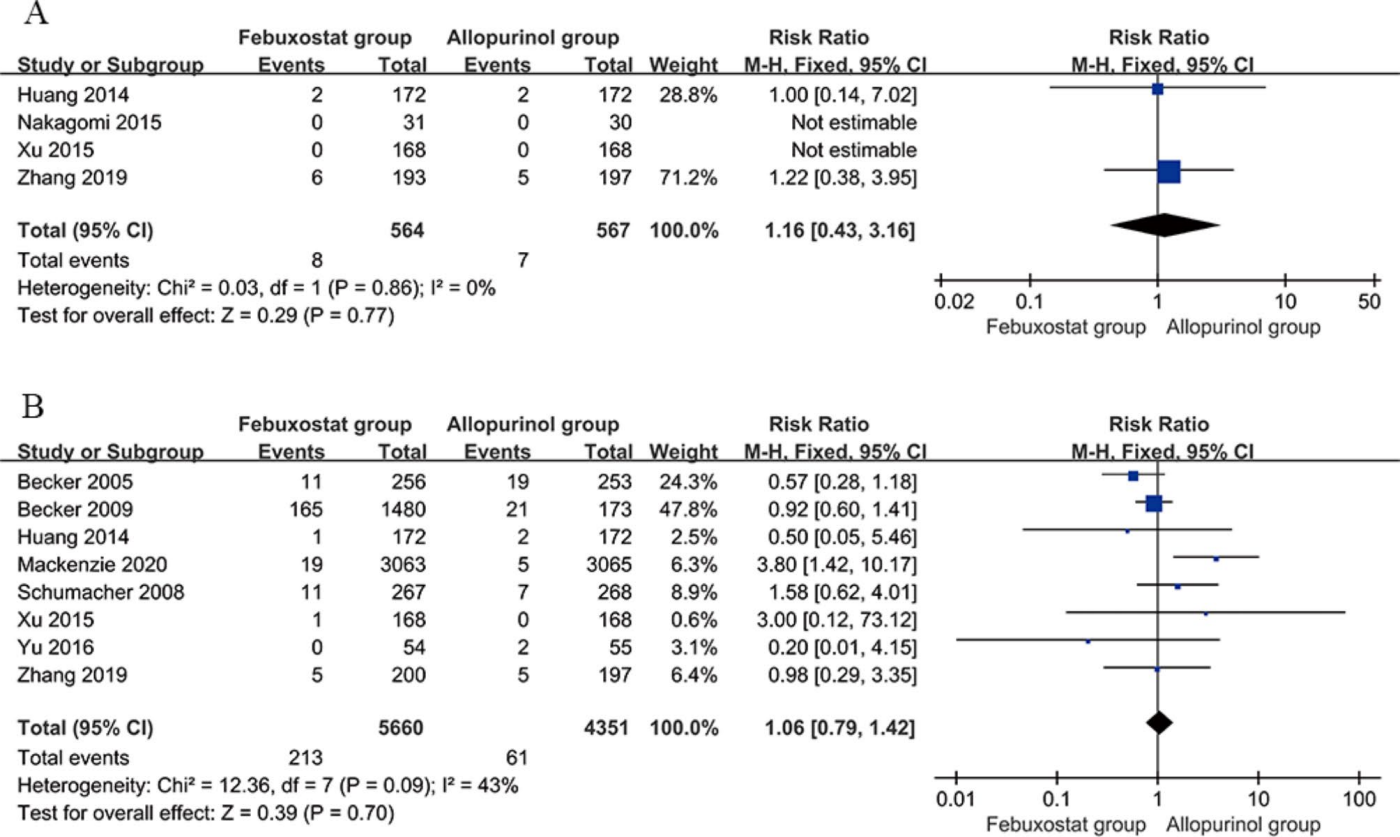
Forest plot comparing the incidence of serious adverse reactions between the febuxostat and allopurinol groups. **a** Febuxostat (40 mg/d) versus allopurinol (200–300 mg/d). **b** Febuxostat (80 mg/d) versus allopurinol (200–300 mg/d). CI, confidence interval

Four studies [21, 24, 26, 28] compared the incidence of serious adverse reactions between the febuxostat group (40 mg/d) and the allopurinol group (200–300 mg/d). The incidence was 8/564 (1.4%) in febuxostat group (40 mg/d) and 7/567 (1.2%) in the allopurinol group (200–300 mg/d), and there was no significant heterogeneity between the studies (*P* = 0.86, I^2^ = 0%). Meta-analysis was conducted using the fixed effects model, and the results indicated that the difference in the incidence of serious adverse reactions between the febuxostat group (40 mg/d) and allopurinol group (200–300 mg/d) was nonsignificant [RR = 1.16; 95% CI (0.43, 3.16), *P* = 0.77]. Figure 6A presents the details of the meta-analysis.

Eight studies [18, 19, 21, 23, 25–28] compared the incidence of serious adverse reactions between the febuxostat group (80 mg/d) and allopurinol group (200–300 mg/d). The incidence of serious adverse reactions was 213/5660 (3.7%) in febuxostat group (80 mg/d) and 61/4351 (1.4%) in the allopurinol group (200–300 mg/d), and there was no significant heterogeneity across the studies (*P* = 0.09, I^2^ = 43%). Meta-analysis was conducted using the fixed effects model, and the results indicated that the difference in the incidence of serious adverse reactions between the febuxostat group (80 mg/d) and allopurinol group (200–300 mg/d) was nonsignificant [RR = 1.06; 95% CI (0.79, 1.42), *P* = 0.70]. Figure 6B presents the details of the meta-analysis.

#### Incidence of adverse cardiovascular reactions

**Fig. 7:**
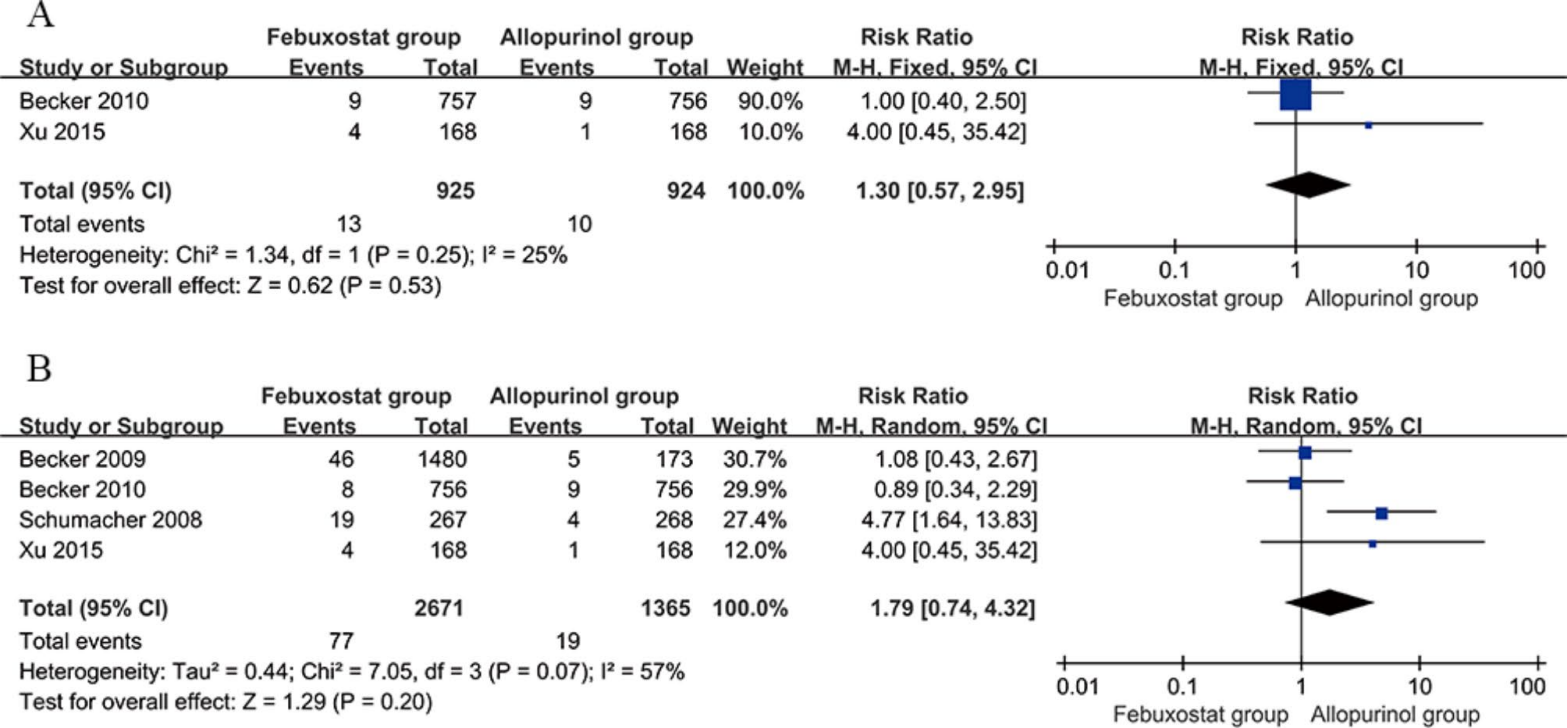
Forest plot comparing the incidence of adverse cardiovascular reactions between the febuxostat and allopurinol groups. **a** Febuxostat (40 mg/d) versus allopurinol (200–300 mg/d). **b** Febuxostat (80 mg/d) versus allopurinol (200–300 mg/d). CI, confidence interval

Two studies [20, 26] compared the incidence of adverse cardiovascular reactions between the febuxostat group (40 mg/d) and the allopurinol group (200–300 mg/d). The incidence was 13/925 (1.4%) in the febuxostat group (40 mg/d) and 10/924 (1.1%) in the allopurinol group (200–300 mg/d), and there was no significant heterogeneity across studies (*P* = 0.25, I^2^ = 25%). Meta-analysis was conducted using the fixed effects model, and the results indicated that the difference in the incidence of serious adverse cardiovascular reactions between the febuxostat group (40 mg/d) and allopurinol group (200–300 mg/d) was nonsignificant [RR = 1.30; 95% CI (0.57, 2.95), *P* = 0.53]. Figure 7A presents the details of the meta-analysis.

Four studies [19, 20, 25, 26] compared the incidence of adverse cardiovascular reactions in the febuxostat group (80 mg/d) and the allopurinol group (200–300 mg/d). The incidence was 77/2671 (2.9%) in the febuxostat group (80 mg/d) and 19/1365 (1.4%) in the allopurinol group (200–300 mg/d), and there was significant heterogeneity across studies (*P* = 0.07, I^2^ = 57%). Meta-analysis was conducted using the random effects model, and the results indicated that the difference in the incidence of serious adverse cardiovascular reactions between the febuxostat group (80 mg/d) and allopurinol group (200–300 mg/d) was nonsignificant [RR = 1.79; 95% CI (0.74, 4.32), *P* = 0.20]. Sensitivity analysis revealed no directional changes in the effects after excluding studies one at a time, thus indicating that the findings of the study were relatively stable. Figure 7B presents the details of the meta-analysis.

### GRADE evidence quality assessment

The quality of evidence for the eight outcome indicators was evaluated using the GRADE approach, and the specific results are presented in Table 3. The main reasons for downgrading included the absence of blinding in the included studies, inadequate allocation concealment, substantial heterogeneity among some studies, and confidence intervals crossing the threshold for clinical decision-making, which decreased the scientific rigor of the study methods and the reliability of the research findings.

**Table 3:** GRADE assessment of outcome indicators

Outcome indicators	Risk of bias	Inconsistency	Indirectness	Imprecision	Publication bias	GRADE quality
Febuxostat (40 mg/d) versus allopurinol (200–300 mg/d) in percentage of patients achieving serum uric acid levels 6.0 mg/dL or less	Serious	Serious	Not serious	Not serious	Undetected	⊕⊕○○/ Low
Febuxostat (80 mg/d) versus allopurinol (200–300 mg/d) in percentage of patients achieving serum uric acid levels 6.0 mg/dL or less	Serious	Serious	Not serious	Not serious	Undetected	⊕⊕○○/ Low
Febuxostat (40 mg/d) versus allopurinol (200–300 mg/d) in incidence of gout	Serious	Not serious	Not serious	Not serious	Undetected	⊕⊕○/Moderate
Febuxostat (80 mg/d) versus allopurinol (200–300 mg/d) in incidence of gout	Serious	Serious	Not serious	Not serious	Undetected	⊕⊕○○/ Low
Febuxostat (40 mg/d) versus allopurinol (200–300 mg/d) in incidence of serious adverse reactions	Serious	Not serious	Not serious	Not serious	Undetected	⊕⊕○/Moderate
Febuxostat (80 mg/d) versus allopurinol (200–300 mg/d) in incidence of serious adverse reactions	Serious	Serious	Not serious	Not serious	Undetected	⊕⊕○○/ Low
Febuxostat (40 mg/d) versus allopurinol (200–300 mg/d) in incidence of adverse cardiovascular reactions	Serious	Not serious	Not serious	Not serious	Undetected	⊕⊕○/Moderate
Febuxostat (80 mg/d) versus allopurinol (200–300 mg/d) in incidence of adverse cardiovascular reactions	Serious	Serious	Not serious	Not serious	Undetected	⊕⊕○○/ Low

GRADE, Grading of Recommendations Assessment, Development, and Evaluation

## Discussion

Hyperuricemia has become more prevalent in younger people in recent years [37]. The prevalence of hyperuricemia is rising steadily because of people’s changing lifestyles and better living standards [38]. The most common clinical manifestation of hyperuricemia is gout, which seriously affects the mental and physical health of patients and impacts their quality of life [39]. Hyperuricemia can induce many major diseases, such as coronary heart disease, myocardial infarction, diabetes, hyperlipidaemia, metabolic syndrome, and chronic kidney disease [40, 41]. Therefore, hyperuricemia is regarded as a metabolic disease that significantly affects the daily lives of people and threatens their health. Effectively lowering levels of uric acid is key to reducing the risk of gout and preventing the occurrence and development of comorbidities [42]. Most patients need long-term or even lifelong treatment with urate-lowering drugs; however, long-term use of those drugs has certain side effects [43]. Therefore, it is of great significance both for clinical practices and public health to actively seek safe and effective strategies to prevent and treat high levels of uric acid.

Allopurinol and febuxostat are the main drugs that are generally used for inhibiting uric acid synthesis in clinical practice, but their efficacy and safety are still controversial [44]. This meta-analysis differs from previous similar meta-analyses in its specific emphasis on the impact of dosage on efficacy and safety. Moreover, the use of the GRADE rating system allows for the identification and evaluation of the limitations of the existing evidence. This focus helps provide more specific and detailed clinical practice guidance. Eleven randomized controlled trials were included in the meta-analysis to compare the effectiveness and safety of varying doses of febuxostat and allopurinol, and the results offer a theoretical basis and guidance for clinical treatment with drugs. Allopurinol, which was developed and marketed in the 1960s, is a widely used first-line inhibitor of uric acid synthesis in clinical practice [45]. Allopurinol and its metabolite oxypurinol can inhibit reduced xanthine oxidase and prevent hypoxanthine and xanthine from metabolizing to uric acid, thereby reducing uric acid synthesis [46, 47]. Allopurinol has a single target, whereas febuxostat inhibits the oxidized and reduced forms of xanthine oxidase [48]. Febuxostat could be metabolized by the liver, and there is no need to adjust the dosage levels of this drug in patients with mild or moderate renal insufficiency; thus, it can be used in patients with allopurinol allergy and chronic renal insufficiency [49, 50]. The meta-analysis results implied that the percentage of patients achieving serum uric acid levels of 6.0 mg/dL or less was comparable between the febuxostat group (40 mg/d) and allopurinol group (200–300 mg/d), but the percentage of patients achieving serum uric acid levels of 6.0 mg/dL or less was significantly higher in the febuxostat group (80 mg/d) than in the allopurinol group (200–300 mg/d), indicating that febuxostat (80 mg/d) was more effective in lowering uric acid than allopurinol (200–300 mg/d), This finding may be due to febuxostat having a mechanism that involves inhibiting both oxidized and reducing xanthine oxidases and higher selectivity for xanthine oxidase and activity, thus, febuxostat is more effective in reducing uric acid synthesis than allopurinol. A significant proportion of the febuxostat and allopurinol groups did not achieve their target serum urate levels; therefore, it can be considered to use combined urate-lowering therapies for treatment. However, both febuxostat (40 mg/d) and febuxostat (80 mg/d) were comparable to allopurinol (200–300 mg/d) with respect to reducing the incidence of gout. The reason that febuxostat was not found to be effective in reducing the incidence of gout attacks could also be due to the short duration of the included studies and some studies included patients with and without gout.

In addition to pursuing therapeutic efficacy during treatment, adverse reactions should also be considered. Many clinical applications have found that allopurinol is prone to cause more serious adverse reactions [51]. After entering the body, allopurinol is metabolized in the liver to active hydroxypurinol, which is excreted by the kidneys and tends to accumulate in patients with renal insufficiency, thereby increasing the risk of drug poisoning [52]. Long-term use of allopurinol can cause leukopenia and thrombocytopenia, eosinophilia, fever, severe erythema multiforme, toxic epidermal necrolysis (TEN), and in severe cases, even secondary infection or internal organ failure and endanger the lives of the patients [53, 54]. The common adverse reactions of febuxostat mainly include abnormal liver function, diarrhea, headache, nausea, vomiting, and rash [55, 56]. The findings of the meta-analysis indicated that the differences in serious adverse reactions between the febuxostat group (40 mg/d) and the allopurinol group (200–300 mg/d) or between the febuxostat group (80 mg/d) and the allopurinol group (200–300 mg/d) were nonsignificant, but the incidence was 1.4% in the febuxostat group (40 mg/d) and 3.7% in the febuxostat group (80 mg/d). Therefore, serious adverse reactions should be closely observed and treated in time when increasing febuxostat doses during treatment. Febuxostat has previously been shown to increase adverse cardiovascular reactions and is recommended to be used with caution in patients with cardiovascular and cerebrovascular diseases [57]. In this meta-analysis, neither the febuxostat group (40 mg/d) nor the febuxostat group (80 mg/d) had an increased risk of cardiovascular accidents compared with the allopurinol group (200–300 mg/d), but it was also seen that the incidence of adverse cardiovascular reactions increased from 1.4 to 2.9% with an increasing dose of febuxostat from 40 mg to 80 mg. Therefore, it is also recommended that we pay attention to adverse cardiovascular reactions when increasing doses of febuxostat during treatment. There was significant variation in the duration of follow-up among the different studies. In some studies, the follow-up period may be too short, such as 12–16 weeks, which might not be sufficient to evaluate cardiovascular mortality. The relatively short duration of follow-up may limit a comprehensive assessment of endpoints such as cardiovascular mortality. The conclusion of this meta-analysis still needs to be verified by larger-sample, multicentre, rigorously designed high-quality clinical randomized controlled trials due to the small sample size and short duration of follow-up in some studies.

The meta-analysis had a few limitations. (1) A few RCTs did not include allocation concealment and blinding, resulting in a higher selection risk and implementation and measurement bias and thus affecting the accuracy of the results. (2) The follow-up durations were inconsistent, which may lead to heterogeneity in results; in some studies, the follow-up duration was too short, which might not be sufficient to evaluate cardiovascular mortality. (3) Only 5 common English databases were searched, and studies published in other languages were missed. (4) Some of outcome indicators were not examined in enough studies to perform subgroup analysis. (5) Only serious adverse reactions, such as cardiovascular reactions, were analyzed, while common adverse reactions (such as liver function damage or skin-related adverse reactions) were not analyzed. (6) Placebo was not included in this meta-analysis. (7) The studies included arose from vastly different populations.

In conclusion, febuxostat (80 mg/d) was associated with a higher percentage of patients achieving serum uric acid levels of 6.0 mg/dL or less than than with allopurinol (200–300 mg/d); however, it did not show better efficacy in reducing the incidence of gout. Close attention to adverse reactions was recommended when increasing the doses, although the incidence of serious reactions and adverse cardiovascular reactions was comparable between the different doses of febuxostat vs. allopurinol in this study. The conclusion of this study still needs to be validated by further clinical RCTs with larger sample sizes, multicentre data sources, rigorously designed protocols, and high quality.

### Electronic supplementary material

Below is the link to the electronic supplementary material.

Supplementary Material 1

Supplementary Material 2

## Data Availability

All data generated or analysed during this study are included in this published article [and its supplementary information files.
