# Supplementary material for: Effectiveness and safety of different doses of febuxostat compared with allopurinol in the treatment of hyperuricemia: a meta-analysis of randomized controlled trials
Source: BMC Pharmacol Toxicol. 2023 Dec 14;24:79. doi: 10.1186/s40360-023-00723-5 (PMC10722766; doi:10.1186/s40360-023-00723-5)
Supplement: Supplementary file 2 — Supplementary Material 2 [file 40360_2023_723_MOESM2_ESM.docx]

**1. The search strategy for Cochrane.**

#1: 'Hyperuricemia':ti,ab OR 'gout':ti,ab OR 'uric acid':ti,ab OR 'SUA':ti,ab

#2: 'Febuxostat':ti,ab OR '6720, TEI':ti,ab OR 'TEI-6720':ti,ab OR 'TEI6720 Uloric':ti,ab

#3: 'Allopurinol':ti,ab OR 'Uribenz':ti,ab OR 'Allopurin':ti,ab OR 'Allorin':ti,ab OR 'Allpargin':ti,ab OR 'Allural':ti,ab OR 'Pan Quimica':ti,ab OR 'Apulonga':ti,ab OR 'Apurin':ti,ab OR 'Atisuril':ti,ab OR 'Bleminol':ti,ab OR 'Caplenal':ti,ab OR 'Capurate':ti,ab OR 'Cellidrin':ti,ab OR 'Embarin':ti,ab OR 'Suspendol':ti,ab OR 'Foligan':ti,ab OR 'Hamarin':ti,ab OR 'Lopurin':ti,ab OR 'Lysuron':ti,ab OR 'Jenapurinol':ti,ab OR 'Milurit':ti,ab OR 'Milurite':ti,ab OR 'Novopurol':ti,ab OR 'Uripurinol':ti,ab OR 'Urosin':ti,ab OR 'Urtias':ti,ab OR 'Xanthomax':ti,ab OR 'Uridocid':ti,ab OR 'Xanturic':ti,ab OR 'Zygout':ti,ab OR 'Zyloprim':ti,ab OR 'Zyloric':ti,ab OR 'Pureduct':ti,ab OR 'Purinol':ti,ab OR 'Progout':ti,ab OR 'Remid':ti,ab OR 'Rimapurinol':ti,ab OR 'Roucol':ti,ab OR 'Tipuric':ti,ab OR 'Allohexal':ti,ab OR 'Allohexan':ti,ab OR 'Alloprin':ti,ab

#4: #1 AND #2 AND #3

**2. The search strategy for Embase.**

#1: (Hyperuricemia):ti,ab OR (gout):ti,ab OR (uric acid):ti,ab OR (SUA):ti,ab

#2: (Febuxostat):ti,ab OR (6720, TEI):ti,ab OR (TEI-6720):ti,ab OR (TEI6720 Uloric):ti,ab

#3: (Allopurinol):ti,ab OR (Uribenz):ti,ab OR (Allopurin):ti,ab OR (Allorin):ti,ab OR (Allpargin):ti,ab OR (Allural):ti,ab OR (Pan Quimica):ti,ab OR (Apulonga):ti,ab OR (Apurin):ti,ab OR (Atisuril):ti,ab OR (Bleminol):ti,ab OR (Caplenal):ti,ab OR (Capurate):ti,ab OR (Cellidrin):ti,ab OR (Embarin):ti,ab OR (Suspendol):ti,ab OR (Foligan):ti,ab OR (Hamarin):ti,ab OR (Lopurin):ti,ab OR (Lysuron):ti,ab OR (Jenapurinol):ti,ab OR (Milurit):ti,ab OR (Milurite):ti,ab OR (Novopurol):ti,ab OR (Uripurinol):ti,ab OR (Urosin):ti,ab OR (Urtias):ti,ab OR (Xanthomax):ti,ab OR (Uridocid):ti,ab OR (Xanturic):ti,ab OR (Zygout):ti,ab OR (Zyloprim):ti,ab OR (Zyloric):ti,ab OR (Pureduct):ti,ab OR (Purinol):ti,ab OR (Progout):ti,ab OR (Remid):ti,ab OR (Rimapurinol):ti,ab OR (Roucol):ti,ab OR (Tipuric):ti,ab OR (Allohexal):ti,ab OR (Allohexan):ti,ab OR (Alloprin):ti,ab

#4: #1 AND #2 AND #3

**3. The search strategy for PubMed.**

#1: "Hyperuricemia"[Mesh]

#2: gout[Title/Abstract] OR uric acid[Title/Abstract] OR SUA[Title/Abstract]

#3: #1 OR #2

#4: "Febuxostat"[Mesh]

#5: TEI 6720[Title/Abstract] OR 6720, TEI[Title/Abstract] OR TEI-6720[Title/Abstract] OR TEI6720 Uloric[Title/Abstract] OR 2-’3-cyano-4-isobutoxyphenyl)-4-methyl-5-thiazolecarboxylic acid[Title/Abstract]

#6: #4 OR #5

#7: "Allopurinol"[Mesh]

#8: Uribenz[Title/Abstract] OR Allopurin[Title/Abstract] OR Allorin[Title/Abstract] OR Allpargin[Title/Abstract] OR Allural[Title/Abstract] OR Pan Quimica[Title/Abstract] OR Apulonga[Title/Abstract] OR Apurin[Title/Abstract] OR Atisuril[Title/Abstract] OR Bleminol[Title/Abstract] OR Caplenal[Title/Abstract] OR Capurate[Title/Abstract] OR Cellidrin[Title/Abstract] OR Embarin[Title/Abstract] OR Suspendol[Title/Abstract] OR Foligan[Title/Abstract] OR Hamarin[Title/Abstract] OR Lopurin[Title/Abstract] OR Lysuron[Title/Abstract] OR Jenapurinol[Title/Abstract] OR Milurit[Title/Abstract] OR Milurite[Title/Abstract] OR Novopurol[Title/Abstract] OR Uripurinol[Title/Abstract] OR Urosin[Title/Abstract] OR Urtias[Title/Abstract] OR Xanthomax[Title/Abstract] OR Uridocid[Title/Abstract] OR Xanturic[Title/Abstract] OR Zygout[Title/Abstract] OR Zyloprim[Title/Abstract] OR Zyloric[Title/Abstract] OR Pureduct[Title/Abstract] OR Purinol[Title/Abstract] OR Progout[Title/Abstract] OR Remid[Title/Abstract] OR Rimapurinol[Title/Abstract] OR Roucol[Title/Abstract] OR Tipuric[Title/Abstract] OR Allohexal[Title/Abstract] OR Allohexan[Title/Abstract] OR Alloprin[Title/Abstract]

#9: #7 OR #8

#10: #3 AND #6 AND #9

**4. The search strategy for Web of Science.**

#1:TS=(Hyperuricemia OR gout OR uric acid OR SUA)

#2:TS=(Febuxostat OR 6720, TEI OR TEI-6720 OR TEI6720 Uloric)

#3: TS= (Allopurinol OR Uribenz OR Allopurin OR Allorin OR Allpargin OR Allural OR Pan Quimica OR Apulonga OR Apurin OR Atisuril OR Bleminol OR Caplenal OR Capurate OR Cellidrin OR Embarin OR Suspendol OR Foligan OR Hamarin OR Lopurin OR Lysuron OR Jenapurinol OR Milurit OR Milurite OR Novopurol OR Uripurinol OR Urosin OR Urtias OR Xanthomax OR Uridocid OR Xanturic OR Zygout OR Zyloprim OR Zyloric OR Pureduct OR Purinol OR Progout OR Remid OR Rimapurinol OR Roucol OR Tipuric OR Allohexal OR Allohexan OR Alloprin)

#4: #1 AND #2 AND #3

**5. The search strategy for ClinicalTrials.gov.**

#1: (Hyperuricemia OR gout OR uric acid OR SUA)

#2: (Febuxostat OR 6720, TEI OR TEI-6720 OR TEI6720 Uloric) AND (Allopurinol OR Uribenz OR Allopurin OR Allorin OR Allpargin OR Allural OR Pan Quimica OR Apulonga OR Apurin OR Atisuril OR Bleminol OR Caplenal OR Capurate OR Cellidrin OR Embarin OR Suspendol OR Foligan OR Hamarin OR Lopurin OR Lysuron OR Jenapurinol OR Milurit OR Milurite OR Novopurol OR Uripurinol OR Urosin OR Urtias OR Xanthomax OR Uridocid OR Xanturic OR Zygout OR Zyloprim OR Zyloric OR Pureduct OR Purinol OR Progout OR Remid OR Rimapurinol OR Roucol OR Tipuric OR Allohexal OR Allohexan OR Alloprin)

#3: (Allopurinol OR Uribenz OR Allopurin OR Allorin OR Allpargin OR Allural OR Pan Quimica OR Apulonga OR Apurin OR Atisuril OR Bleminol OR Caplenal OR Capurate OR Cellidrin OR Embarin OR Suspendol OR Foligan OR Hamarin OR Lopurin OR Lysuron OR Jenapurinol OR Milurit OR Milurite OR Novopurol OR Uripurinol OR Urosin OR Urtias OR Xanthomax OR Uridocid OR Xanturic OR Zygout OR Zyloprim OR Zyloric OR Pureduct OR Purinol OR Progout OR Remid OR Rimapurinol OR Roucol OR Tipuric OR Allohexal OR Allohexan OR Alloprin)

#4: #1 AND #2 AND #3
